# Stroke neurovascular responses to exercise: A novel rehabilitation paradigm

**DOI:** 10.1113/EP093544

**Published:** 2026-06-17

**Authors:** Alicen A. Whitaker‐Hilbig, Allison S. Hyngstrom, Matthew J. Durand

**Affiliations:** ^1^ Department of Anesthesiology Medical College of Wisconsin Milwaukee Wisconsin USA; ^2^ Cardiovascular Research Center Medical College of Wisconsin Milwaukee Wisconsin USA; ^3^ Department of Physical Therapy Marquette University Milwaukee Wisconsin USA; ^4^ Department of Physiology Medical College of Wisconsin Milwaukee Wisconsin USA

**Keywords:** exercise physiology, microvascular health, neurorehabilitation, stroke, vascular blood flow

## Abstract

Stroke is a leading cause of long‐term disability, impacting the cerebrovascular, peripheral vascular and neuromotor systems. Characterising vascular responses to exercise post‐stroke offers valuable insights into the impairments that might influence outcomes during neurorehabilitation. We have shown a blunted cerebrovascular response to moderate‐intensity continuous exercise and high‐intensity interval exercise in individuals post‐stroke compared with control subjects. We have also shown an inability of the peripheral microvasculature to vasodilate in response to movement, potentially contributing to an attenuated peripheral blood flow response to submaximal and maximal isometric quadricep contractions. Reduced blood flow responses to exercise within both the cerebrovascular and peripheral vascular systems might thus have detrimental effects on the downstream tissues, influencing neuromotor function and stroke recovery. By identifying the exact mechanisms contributing to poor cerebrovascular and peripheral vascular responses to exercise, we can implement targeted interventions, such as mitochondria‐targeted antioxidants and/or ischaemic conditioning. Leveraging the interdependent relationship between vascular health and neuromotor function might optimise post‐stroke rehabilitation and accelerate functional recovery.

## Abstract (highlights)

- **What is the topic of this review** **?**
  After stroke, reduced blood flow responses to exercise are found within both the cerebrovascular and peripheral vascular systems, which can have detrimental effects on downstream tissues (neurons, skeletal muscle, etc.), potentially limiting exercise capacity and clinical outcomes.
- **What advances does it highlight?**
  By identifying mechanism‐based interventions that target both the vascular and neuromotor systems, we can leverage their interdependent relationship to generate synergistic improvements in physical function.

## 1 INTRODUCTION

In the USA, stroke is the leading cause of long‐term disability, with approximately half a million individuals requiring ongoing rehabilitation after hospital discharge each year (American Heart Association, 2023). Although stroke is most commonly characterised by deficits in neuromotor function, the underlying pathology originates within the cerebrovascular system. Nevertheless, vascular health remains an underappreciated component of both neurorehabilitation and long‐term management strategies for physical function after stroke. It is important to understand how the vascular responses to exercise differ in individuals post‐stroke, given evidence of impaired flow‐mediated dilatation (FMD; Bartsch et al., 2024), reduced carbon dioxide reactivity (Maeda et al., 1993), impaired neurovascular coupling (Ferreira et al., 2025), compromised cardiac output (McNamara et al., 2024) and heightened sympathetic drive (Barkhudaryan et al., 2025) after stroke. Reduced blood flow during exercise might compromise the delivery of oxygen and glucose and hinder the clearance of metabolic byproducts, thereby limiting neuronal activity, skeletal muscle contractions and the restoration of physical function after a stroke.

## 2 CEREBROVASCULAR RESPONSE TO EXERCISE POST‐STROKE

**FIGURE 1:**
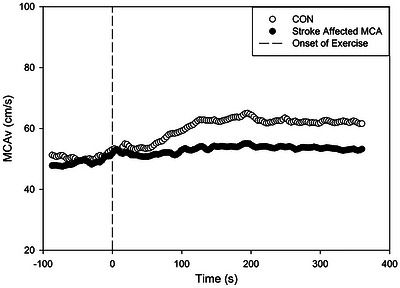
MCAv kinetic response in CON (*n* = 18) and stroke‐affected middle cerebral artery (*n* = 14). Abbreviations: CON, control participants; MCA, middle cerebral artery; MCAv, middle cerebral artery velocity. This figure has been previously published as Kempf KS, Whitaker AA, Lui Y, Witte E, Perdomo SJ, Ward JL, Eickmeyer S, Ledbetter L, Abraham M, & Billinger SA. (2019). The effect of stroke on middle cerebral artery blood flow velocity dynamics during exercise. *Journal of Neurologic Physical Therapy*, 43(4), 212–219. The Creative Commons license does not apply to this content. Use of the material in any format is prohibited without written permission from the publisher, Wolters Kluwer Health, Inc. Copyright © 2019 Academy of Neurologic Physical Therapy, APTA. Reprinted by Permission of Wolters Kluwer Health, Inc.

Given that stroke originates within the cerebrovascular system, it is essential to characterise the response to currently recommended exercise prescriptions, such as moderate‐intensity exercise (45%–55% of heart rate reserve). In individuals 3 months post‐stroke, we found a blunted middle cerebral artery blood velocity (MCAv) response in comparison to age‐ and sex‐matched control subjects (Figure 1; Kempf et al., 2019). Previous studies have prescribed exercise intensity based on measures of the gas exchange threshold and maximal oxygen consumption to ensure the absence of hypocapnia‐induced decreases in MCAv (Weston et al., 2022), whereas we used a clinically relevant prescription of moderate‐intensity exercise that accounts for underprescribing intensity owing to elevated resting heart rates in individuals post‐stroke. Importantly, we found that end‐tidal carbon dioxide increased from baseline to moderate‐intensity exercise steady state and did not differ between groups (Kempf et al., 2019).

Interestingly, individuals 3 months post‐stroke exhibited substantial interindividual variability in their MCAv response to moderate‐intensity exercise, prompting further investigation into characteristics that might influence this response. In comparison to non‐responders, individuals post‐stroke who responded to the exercise (increased MCAv by ≥2 cm/s from baseline during steady state) had significantly greater estimated aerobic fitness and higher levels of physical activity, measured via the non‐exercise estimated maximal oxygen consumption questionnaire (Billinger et al., 2021). Additionally, the same individuals at 6 months post‐stroke who responded to the exercise also walked significantly farther during the 6‐min walk test, potentially supporting our hypothesis that the cerebrovascular response to exercise might contribute to physical function post‐stroke. However, additional research is warranted to confirm the association between maximal oxygen consumption measured during a graded maximal exercise test and the MCAv response post‐stroke and to clarify the causal relationship with lower physical function after stroke. Further investigation to determine the reliability of classifying individuals as MCAv ‘responders’ or ‘non‐responders’ to moderate‐intensity exercise based on a change score of ≥2 cm/s is also warranted, in addition to the potential utility of threshold values based on absolute or percentage changes in MCAv.

Given that moderate‐intensity aerobic exercise proved to be challenging only for some individuals, we wanted to examine the cerebrovascular response to a more demanding exercise stimulus, called high‐intensity interval exercise (HIIE). HIIE represents a substantial physiological challenge, not only because cardiovascular intensity exceeds the anaerobic threshold, but also owing to the rapid and repetitive transitions. Despite this, HIIE has gained popularity in stroke neurorehabilitation owing to the shorter amount of time needed to improve aerobic fitness (Moncion et al., 2024) and walking function (Boyne et al., 2023) in comparison to moderate‐intensity continuous exercise.

**FIGURE 2:**
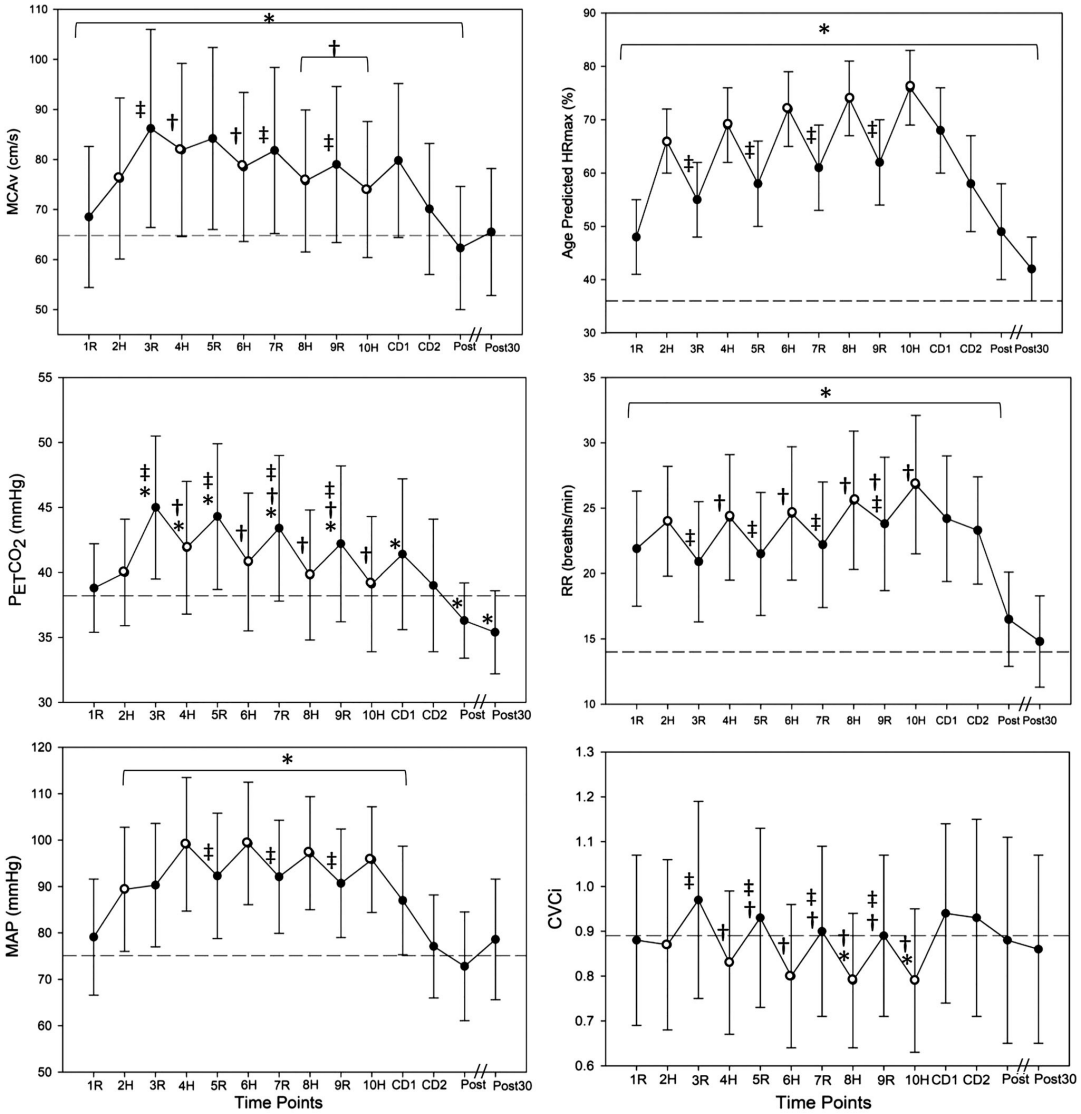
Response to high‐intensity interval exercise (HIIT, *n* = 24); horizontal dashed line represents baseline value. *Significantly different from baseline (*P* < 0.005). †Significantly different from minute 3 (MCAv peak). ‡Significantly different from previous high‐intensity bout. Parameters are as follows: middle cerebral artery blood velocity (MCAv; in centimetres per second), age‐predicted maximal heart rate (HRmax), expired end‐tidal carbon dioxide (P_ET_CO_2_; in millimetres of mercury; *n* = 23), respiratory rate (RR; in breaths per minute; *n* = 23), mean arterial pressure (MAP; in millimetres of mercury) and cerebrovascular conductance index (CVCi; MCAv/MAP). Abbreviations: CD, cool down; H, high intensity (open dots); post, immediately following HIIT; post30, 30 min after HIIT; R, active recovery. This figure has been previously published as an open access article and is reprinted here without adaptation. Whitaker AA, Aaron SE, Kaufman CS, Kurtz BK, Bai SX, Vidoni ED, Montgomery RN, & Billinger SA. (2022). Cerebrovascular response to an acute bout of low‐volume high‐intensity interval exercise and recovery in young healthy adults. *Journal of Applied Physiology*, 132(1), 236–246.

**FIGURE 3:**
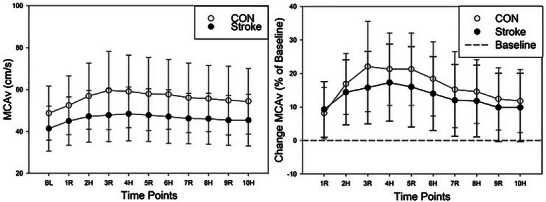
Cerebrovascular response to high‐intensity interval exercise in individuals post‐stroke and control subjects. Controls (CON) = open circles. Stroke ipsilesional hemisphere = filled circles. Abbreviations: BL, baseline; H, high‐intensity; MCAv, middle cerebral artery blood velocity; R, active recovery. This figure has been published previously as Whitaker AA, Waghmare S, Montgomery RN, Aaron SE, Eickmeyer SM, Vidoni ED, & Billinger SA. (2023). Lower middle cerebral artery blood velocity during low‐volume high‐intensity interval exercise in chronic stroke. *Journal of Cerebral Blood Flow & Metabolism*, 271678 × 231201472. Copyright © 2026 by International Society for Cerebral Blood Flow and Metabolism. Reprinted by Permission of Sage Publications.

To determine the ‘normal’ physiological response to HIIE, we initially characterised the MCAv response during 1‐min interval HIIE in young healthy adults (Figure 2; Whitaker et al., 2022). During the onset of HIIE, MCAv increased up to min 3, after which it started to decrease during high‐intensity exercise, probably owing to hyperventilation‐induced reductions in carbon dioxide and subsequent downstream microvascular vasoconstriction. During active recovery, MCAv rebounded as breathing normalised and carbon dioxide levels increased, promoting microvascular vasodilatation. When examining the cerebrovascular response to HIIE in individuals with chronic stroke, we quantified the coefficient of variation between the 1‐min high‐intensity and recovery bouts calculated across the entire HIIE session. In comparison to their age‐ and sex‐matched controls, individuals post‐stroke demonstrated reduced fluctuations in MCAv during HIIE ($\bar{x}$ =  5.16% ± 1.80% vs. $\bar{x}$ = 3.84% ± 1.39%, *P* = 0.01; Figure 3; Whitaker et al., 2023). Notably, lower MCAv responsiveness was moderately associated with greater carotid–femoral arterial stiffness in all individuals (*r* = −0.44), including both chronic stroke survivors and their age‐ and sex‐matched controls. Although cerebrovascular stiffness was not assessed directly, diminished arterial compliance might represent a potential mechanism underlying the blunted MCAv response (Furby et al., 2020).

Additionally, we assessed the ability of the cerebrovascular system to regulate blood flow independently during spontaneous changes in peripheral blood pressure, a mechanism called dynamic cerebral autoregulation (dCA). A 5 min seated rest recording of MCAv and mean arterial pressure was used to calculate dCA parameters, via transfer function analysis, within the very low‐frequency band (0.02–0.07 Hz). Although we found no significant differences in dCA between individuals with chronic stroke and their age‐ and sex‐matched controls at baseline, immediately after HIIE during postexercise hypotension, individuals with chronic stroke had attenuated dCA or a diminished capacity to regulate MCAv in response to changes in mean arterial pressure amplitude (group × time interaction effect, *P* ≤ 0.02; Whitaker et al., 2024). Despite this transient reduction in dCA, participants did not report symptoms of orthostatic hypotension, nor did they demonstrate an altered cerebral pressure–flow relationship during a sit‐to‐stand performed ∼6 min after HIIE. The dCA operates predominantly over frequencies corresponding to temporal responses of ∼5–50 s, whereas the cerebral pressure–flow relationship is assessed on a beat‐to‐beat basis. Therefore, the mechanisms underlying a quick cerebral pressure–flow microvascular vasodilatory response (∼3 s) during a sit‐to‐stand might remain intact immediately following HIIE, whereas the mechanisms contributing to longer‐term dCA might be attenuated. Collectively, these findings indicate that the period immediately following HIIE might constitute a transiently vulnerable window for dCA.

Enhancing cerebral blood flow during exercise in individuals post‐stroke might create an optimal environment for neuronal activation and neuroplasticity (Burley et al., 2016). Future research should aim to elucidate the physiological mechanisms underlying the attenuated MCAv response to exercise in individuals post‐stroke in order to develop targeted therapeutic interventions for neurorehabilitation. For example, individuals with chronic stroke had significantly greater improvements in the MCAv response to 6% carbon dioxide (i.e., carbon dioxide reactivity) after 6 months of vigorous‐intensity aerobic exercise (60%–70% heart rate reserve) performed on a treadmill in comparison to a control group receiving usual care (Ivey et al., 2011). However, whether these improvements in carbon dioxide reactivity translated into an improved MCAv response to exercise or enhanced clinical outcomes is unknown.

There are currently limitations in the ability to measure certain cerebrovascular mechanisms directly. For example, the use of transcranial Doppler ultrasound is currently the best way to measure the beat‐to‐beat cerebrovascular response to exercise temporally. Therefore, MCAv is used as a surrogate measure of cerebral blood flow when assuming a constant diameter. However, because we are unable simultaneously to measure the diameter of the middle cerebral artery (MCA) during exercise using transcranial Doppler ultrasound, changes in MCAv might be under‐ or over‐estimating cerebral blood flow during conditions with large stimuli that are known to change MCA diameter significantly (i.e., end‐tidal carbon dioxide > 8 mmHg; Miller et al., 2019). Although the end‐tidal carbon dioxide changes observed during HIIE were <8 mmHg, we did not measure MCA diameter directly. Additionally, although the individuals post‐stroke included within the study had <50% carotid stenosis, we did not directly measure MCA stenosis or MCA stiffness, which could also influence velocity‐based measures. However, the observed associations between MCAv responsiveness to HIIE and carotid–femoral arterial stiffness sparked interest in investigating peripheral vascular mechanisms. By identifying interventions that improve peripheral vascular health, these approaches might also translate to the cerebrovascular system.

## 3 PERIPHERAL VASCULAR RESPONSE TO MOVEMENT AND EXERCISE POST‐STROKE

**FIGURE 4:**
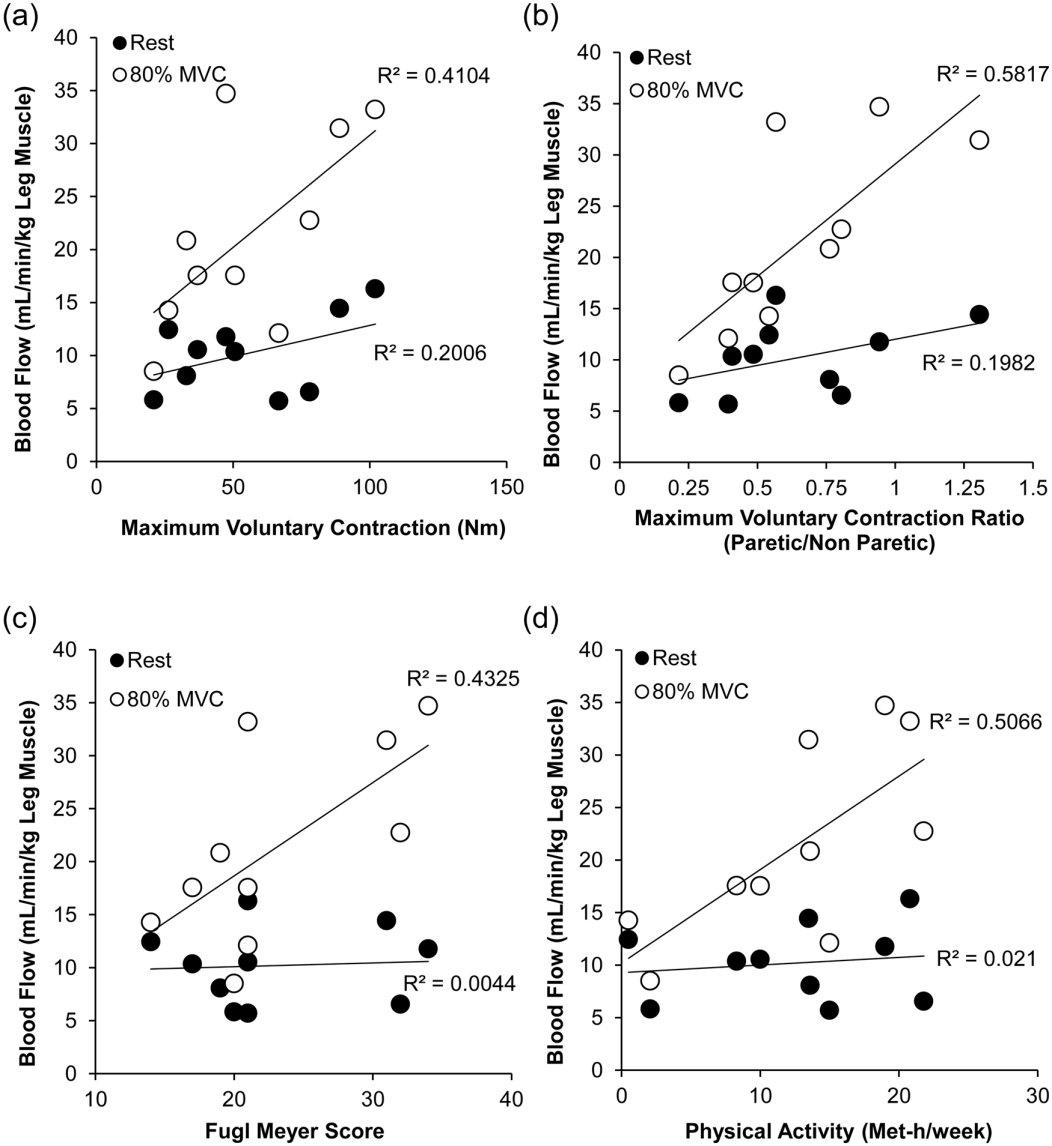
An increased blood flow response in the paretic lower limb following an 80% maximal voluntary contraction (MVC) was positively correlated with: (a) paretic limb strength; (b) symmetry of limb strength; (c) Fugl Meyer score; and (d) physical activity. There were no correlations between any of the measured parameters and paretic lower limb blood flow at rest. This figure has been previously published as an open access article and is reprinted here without adaptation. Durand MJ, Murphy SA, Schaefer KK, Hunter SK, Schmit BD, Gutterman DD, & Hyngstrom AS. (2015). Impaired hyperemic response to exercise post stroke. *PLoS One*, 10(12), e0144023.

Although stroke occurs within the cerebrovascular system, cardiovascular risk factors that contribute to the aetiology of stroke (i.e., atherosclerosis, hypercholesterolaemia, hypertension, etc.) also adversely affect the peripheral vasculature. Secondary hemiparesis and increased sedentary behaviour following stroke also contribute to peripheral vascular dysfunction. Peripheral vascular health is crucial not only for reducing the risk of recurrent stroke, but also for ensuring adequate perfusion of downstream tissues necessary for optimal motor unit activation and muscle contraction (Cole & Brown, 2000; Murphy et al., 2018). Individuals with chronic stroke have reduced paretic leg blood flow during maximal and submaximal isometric exercise (Durand et al., 2015). Reduced paretic leg blood flow during submaximal exercise was associated with decreased quadriceps strength, reduced physical activity and physical impairment (Figure 4). Additionally, reduced hyperaemic blood flow responses to fatiguing exercise were associated with lower motor unit firing rates (Murphy et al., 2019), once again supporting our hypothesis that the vascular response to exercise is essential for neuromotor function. An impaired capacity to increase blood flow in proportion to metabolic demand during exercise might be attributable to impaired large artery FMD, diminished vascular responsiveness to metabolic activity and/or reduced vasodilatory capacity within the downstream microvasculature.

**FIGURE 5:**
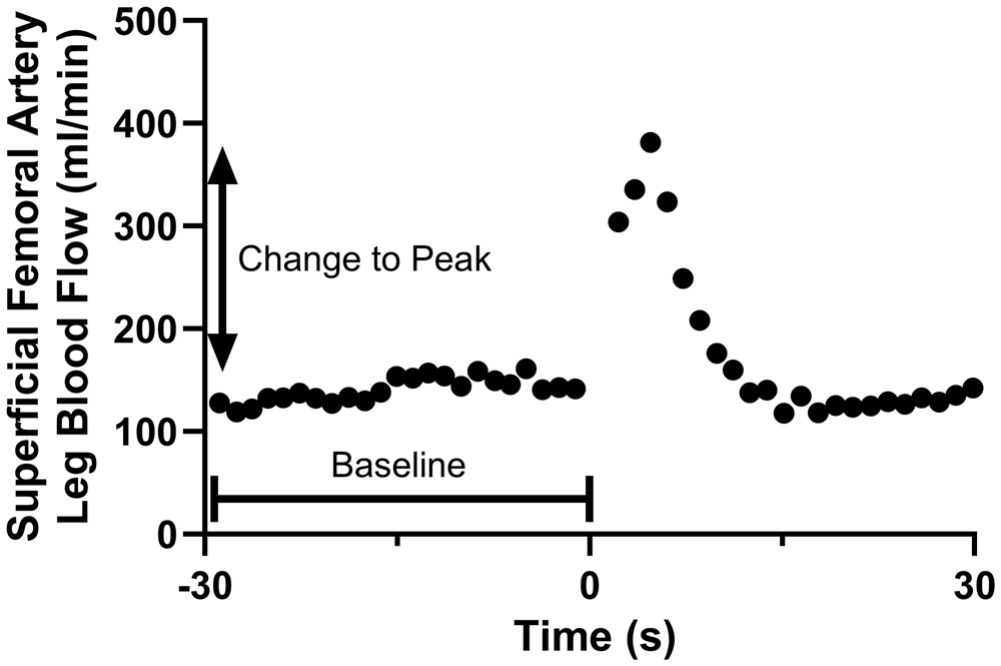
Representative trace of the microvascular leg blood flow response to a single passive limb movement in a young healthy adult.

The microvasculature plays a substantial role in overall vasodilatory capacity and the downstream delivery of oxygen and nutrients. A clinically relevant method for assessing microvascular function is via single passive limb movement (sPLM; Figure 5). This technique evaluates the vasodilatory response to movement independent of increases in skeletal muscle metabolism, by using gravity‐induced increases in shear stress to stimulate microvascular vasodilatation, without eliciting dilatation in large conduit arteries. We have previously published on the reliability of our new methodology that standardises sPLM using a Biodex dynamometer to achieve a consistent 90° movement at 1 Hz, with quadriceps inactivity verified via EMG (Whitaker‐Hilbig et al., 2025). In a recent preliminary findings (Whitaker‐Hilbig et al., 2026), we report a reduced sPLM response in the paretic leg of individuals with chronic stroke in comparison to age‐ and sex‐matched control subjects. Although sPLM appears to be a modest stimulus, the microvasculature must continuously adapt to postural and gravitational changes in daily life, influencing blood flow regulation during activities of daily living, exercise performance and stroke recovery. We found that a diminished microvascular response in the non‐paretic leg was associated with lower maximal quadriceps strength and reduced aerobic fitness, further supporting the premise that microvascular health is integral to physical function following stroke.

In healthy adults, ∼80% of the sPLM response is nitric oxide (NO) mediated (Broxterman et al., 2017). In individuals post‐stroke, a switch from NO‐mediated vasodilatation could be attributable to an increase in reactive oxygen species (ROS) that causes an uncoupling of endothelial NO synthase. ROS also react freely with NO, reducing the amount of NO bioavailability travelling to the vascular smooth muscle for vasodilatation. An early hallmark of microvascular dysfunction is a shift from NO‐mediated vasodilatation to alternative compensatory pathways. Although we are unaware of studies examining the switch from NO specifically within the cerebral vasculature of individuals post‐stroke, previous studies using *ex vivo* arterioles from individuals with coronary artery disease show that when under stress there is a switch from NO to hydrogen peroxide (Freed et al., 2014; Jaramillo‐Torres et al., 2025; Liu et al., 2011), a prevalent form of ROS, being the primary vasodilatory pathway, which, if sustained long‐term, can create an inflammatory environment prone to atherosclerosis (Gutterman et al., 2016). One potential reason for a reduced microvascular response to sPLM in the paretic leg could be attributable to a switch from NO being the primary vasodilatory pathway. However, future research is needed to confirm whether a reduction in the microvascular response to sPLM is attributable to a switch from NO‐mediated vasodilatation or rather an overall reduction in vasodilatory magnitude.

## 4 MECHANISM‐TARGETED INTERVENTIONS FOR VASCULAR HEALTH AND NEUROMOTOR FUNCTION POST‐STROKE

One promising intervention for improving microvascular health is by restoring NO‐mediated vasodilatation using a mitochondria‐targeted antioxidant supplement called MitoQ. Although other non‐specific antioxidants that scavenge cellular ROS have shown little improvements in vascular health, MitoQ increases delivery to mitochondria (the primary source of ROS) by several 100‐fold (Murphy & Smith, 2007). MitoQ has been shown to improve large artery FMD in middle‐aged and older adults, in addition to individuals with peripheral artery disease and chronic kidney disease (Linder et al., 2024; Park et al., 2020; Rossman et al., 2018) after a single acute dose of MitoQ (Park et al., 2020; Rossman et al., 2018) or after chronic supplementation (Kirkman et al., 2023; Rossman et al., 2018). Cutaneous microvascular vasodilatation in response to local heating also trended to a significant improvement (*P* = 0.053) in individuals with chronic kidney disease after 4 weeks of MitoQ supplementation compared with placebo (Kirkman et al., 2023). Our preliminary data in three individuals with chronic stroke show a promising acute increase in brachial artery FMD (∼2%) and NO‐mediated vasodilatation of the cutaneous microvasculature (∼10%) following a single dose of MitoQ compared with a placebo (NCT06930638). MitoQ is also able to cross the blood–brain barrier and is not specific to the vasculature. Therefore, MitoQ might also exert downstream effects on neurons and skeletal muscles, because previous studies have demonstrated improvements in walking capacity in individuals with peripheral artery disease after a single dose of MitoQ (Park et al., 2020), enhanced leg‐extension power in healthy middle‐aged and older adults after 6 weeks of supplementation (Bispham et al., 2017), and greater HIIE training‐induced increases in peak power during graded maximal exercise testing in middle‐aged men following ∼4 weeks of supplementation (Broome et al., 2022). Future studies are needed to investigate the acute and chronic effects of MitoQ supplementation on cerebral and peripheral vascular health, in addition to its potential impact on neuroplasticity and physical function in individuals post‐stroke.

Another potential strategy to enhance microvascular vasodilatation involves ‘preconditioning’ the vessels with an intervention called ischaemic conditioning (IC). During IC, a thigh cuff is placed on the paretic leg and inflated to 250 mmHg to occlude blood flow for 5 min, followed by cuff deflation for 5 min. The repeated inflation and deflation every 5 min for a total of 45 min can precondition the microvasculature for enhanced vasodilatation. Previous studies have shown it to be a whole‐body stimulus causing an increase in vascular, humoral and neurogenic factors. M. J. Durand (vascular biologist) and A. S. Hyngstrom (neurorehabilitation scientist) have previously shown improvements following IC in brachial artery FMD (Hyngstrom et al., 2020), muscle strength (Hyngstrom et al., 2018), motor unit activation (Hyngstrom et al., 2018), walking speed and neuromuscular fatigue (Durand et al., 2019) in chronic stroke. Our recent preliminary findings (Whitaker‐Hilbig et al., 2026) also shows an improvement in the microvascular response to sPLM in the paretic leg of individuals post‐stroke following a single bout of IC, suggesting a potential local therapeutic effect. Although IC shows promising results in early clinical trials investigating 90 day clinical outcomes post‐stroke (Zhao et al., 2023), little is known about the remote effects of IC on specific measures of cerebrovascular health in individuals post‐stroke, such as the cerebrovascular response to exercise. Our promising results within the peripheral vascular system, in addition to positive findings in animal models (Qin et al., 2024), warrant future investigations of the effects of IC on the cerebrovascular system. Further research is also needed to determine the long‐term effects on peripheral and cerebrovascular health, clarify the potential role of IC as an adjunct therapy in post‐stroke neurorehabilitation and identify subgroups most likely to benefit from this intervention (Blauenfeldt et al., 2024).

## 5 CONCLUSION

Microvascular dysfunction is found in both the cerebral and peripheral vascular systems post‐stroke. Reduced blood flow during exercise post‐stroke impairs motor unit activation and strength, potentially limiting exercise capacity and physical recovery. By identifying mechanism‐based interventions that target both microvascular health and neuromotor function, we can leverage their interdependent relationship to generate synergistic improvements in physical function.

## AUTHOR CONTRIBUTIONS

Alicen A. Whitaker‐Hilbig, Allison S. Hyngstrom and Matthew J. Durand have contributed to the conception and design of the work. Alicen A. Whitaker‐Hilbig performed data acquisition, analysis and interpretation. Alicen A. Whitaker‐Hilbig, Allison S. Hyngstrom and Matthew J. Durand contributed to drafting and revising of the work. Alicen A. Whitaker‐Hilbig, Allison S. Hyngstrom and Matthew J. Durand approved the final version of the manuscript and agree to be accountable for all aspects of the work agree to be accountable for all aspects of the work in ensuring that questions related to the accuracy or integrity of any part of the work are appropriately investigated and resolved. All persons designated as authors qualify for authorship, and all those who qualify for authorship are listed.

## CONFLICT OF INTEREST

The authors declare no conflicts of interest.

## Data Availability

Data is available upon reasonable request to the corresponding author.

## References

[eph70345-bib-0001] American Heart Association . (2023). Rehab Therapy After a Stroke, https://www.stroke.org/en/life‐after‐stroke/stroke‐rehab/rehab‐therapy‐after‐a‐stroke

[eph70345-bib-0002] Barkhudaryan, A. , Doehner, W. , & Jauert, N. (2025). Autonomic dysfunction after stroke: An overview of recent clinical evidence and perspectives on therapeutic management. Clinical Autonomic Research, 35(4), 553–563. 40131648 10.1007/s10286-025-01120-0 PMC12325444

[eph70345-bib-0003] Bartsch, B. L. , Hazen, E. M. , Montgomery, R. N. , Trieu, C. , Britton‐Carpenter, A. J. , & Billinger, S. A. (2024). Peripheral vascular function in stroke: Systematic review and meta‐analysis. Journal of Applied Physiology, 136(5), 1182–1194. 38482571 10.1152/japplphysiol.00601.2023 PMC11368525

[eph70345-bib-0004] Billinger, S. A. , Whitaker, A. A. , Morton, A. , Kaufman, C. S. , Perdomo, S. J. , Ward, J. L. , Eickmeyer, S. M. , Bai, S. X. , Ledbetter, L. , & Abraham, M. G. (2021). Pilot study to characterize middle cerebral artery dynamic response to an acute bout of moderate intensity exercise at 3‐ and 6‐months poststroke. Journal of the American Heart Association, 10(3), e017821. 33496192 10.1161/JAHA.120.017821 PMC7955449

[eph70345-bib-0005] Bispham, N. Z. , Santos‐Parker, J. R. , Steward, C. A. C. , Cuevas, L. M. , Rosenberg, H. L. , Murphy, M. P. , Seals, D. R. , & Rossman, M. J. (2017). MitoQ supplementation improves leg‐extension power in healthy late middle‐aged and older adults. The Federation of American Societies for Experimental Biology Journal, 31(1), lb852–lb852.

[eph70345-bib-0006] Blauenfeldt, R. A. , Mortensen, J. K. , Hjort, N. , Valentin, J. B. , Homburg, A.‐M. , Modrau, B. , Sandal, B. F. , Gude, M. F. , Berhndtz, A. B. , Johnsen, S. P. , Hess, D. C. , Simonsen, C. Z. , & Andersen, G. (2024). Effect of remote ischemic conditioning in ischemic stroke subtypes: A post hoc subgroup analysis from the RESIST trial. Stroke; A Journal of Cerebral Circulation, 55(4), 874–879. 10.1161/STROKEAHA.123.046144 PMC10962424 38299363

[eph70345-bib-0007] Boyne, P. , Billinger, S. A. , Reisman, D. S. , Awosika, O. O. , Buckley, S. , Burson, J. , Carl, D. , DeLange, M. , Doren, S. , Earnest, M. , Gerson, M. , Henry, M. , Horning, A. , Khoury, J. C. , Kissela, B. M. , Laughlin, A. , McCartney, K. , McQuaid, T. , Miller, A. , … Dunning, K. (2023). Optimal intensity and duration of walking rehabilitation in patients with chronic stroke: A randomized clinical trial. JAMA Neurology, 80(4), 342–351. 36822187 10.1001/jamaneurol.2023.0033 PMC9951105

[eph70345-bib-0008] Broome, S. C. , Pham, T. , Braakhuis, A. J. , Narang, R. , Wang, H. W. , Hickey, A. J. R. , Mitchell, C. J. , & Merry, T. L. (2022). MitoQ supplementation augments acute exercise‐induced increases in muscle PGC1α mRNA and improves training‐induced increases in peak power independent of mitochondrial content and function in untrained middle‐aged men. Redox Biology, 53, 102341. 35623315 10.1016/j.redox.2022.102341 PMC9142706

[eph70345-bib-0009] Broxterman, R. M. , Trinity, J. D. , Gifford, J. R. , Kwon, O. S. , Kithas, A. C. , Hydren, J. R. , Nelson, A. D. , Morgan, D. E. , Jessop, J. E. , Bledsoe, A. D. , & Richardson, R. S. (2017). Single passive leg movement assessment of vascular function: Contribution of nitric oxide. Journal of Applied Physiology, 123(6), 1468–1476. 28860173 10.1152/japplphysiol.00533.2017 PMC5814686

[eph70345-bib-0010] Burley, C. V. , Bailey, D. M. , Marley, C. J. , & Lucas, S. J. E. (2016). Brain train to combat brain drain; focus on exercise strategies that optimize neuroprotection. Experimental Physiology, 101(9), 1178–1184. 27443587 10.1113/EP085672

[eph70345-bib-0011] Cole, M. A. , & Brown, M. D. (2000). Response of the human triceps surae muscle to electrical stimulation during varying levels of blood flow restriction. European Journal of Applied Physiology, 82(1‐2), 39–44. 10879441 10.1007/s004210050649

[eph70345-bib-0012] Durand, M. J. , Boerger, T. F. , Nguyen, J. N. , Alqahtani, S. Z. , Wright, M. T. , Schmit, B. D. , Gutterman, D. D. , & Hyngstrom, A. S. (2019). Two weeks of ischemic conditioning improves walking speed and reduces neuromuscular fatigability in chronic stroke survivors. Journal of Applied Physiology, 126(3), 755–763. 30653420 10.1152/japplphysiol.00772.2018 PMC6459385

[eph70345-bib-0013] Durand, M. J. , Murphy, S. A. , Schaefer, K. K. , Hunter, S. K. , Schmit, B. D. , Gutterman, D. D. , & Hyngstrom, A. S. (2015). Impaired hyperemic response to exercise post stroke. PLoS ONE, 10(12), e0144023. 26630380 10.1371/journal.pone.0144023 PMC4667998

[eph70345-bib-0014] Ferreira, J. , Alves, F. , Pedro, T. , Fonseca, L. , Gama, G. , Moreira, G. , Azevedo, E. , & Castro, P. (2025). Neurovascular coupling assessment by transcranial doppler in acute stroke could be informative of long‐term cognitive status. Journal of Cerebral Blood Flow and Metabolism, 45(11), 2077–2091. 40615354 10.1177/0271678X251352695 PMC12228646

[eph70345-bib-0015] Freed, J. K. , Beyer, A. M. , LoGiudice, J. A. , Hockenberry, J. C. , & Gutterman, D. D. (2014). Ceramide changes the mediator of flow‐induced vasodilation from nitric oxide to hydrogen peroxide in the human microcirculation. Circulation Research, 115(5), 525–532. 24920698 10.1161/CIRCRESAHA.115.303881 PMC4640193

[eph70345-bib-0016] Furby, H. V. , Warnert, E. A. , Marley, C. J. , Bailey, D. M. , & Wise, R. G. (2020). Cardiorespiratory fitness is associated with increased middle cerebral arterial compliance and decreased cerebral blood flow in young healthy adults: A pulsed ASL MRI study. Journal of Cerebral Blood Flow and Metabolism, 40(9), 1879–1889. 31564194 10.1177/0271678X19865449 PMC7446564

[eph70345-bib-0017] Gutterman, D. D. , Chabowski, D. S. , Kadlec, A. O. , Durand, M. J. , Freed, J. K. , Ait‐Aissa, K. , & Beyer, A. M. (2016). The human microcirculation: Regulation of flow and beyond. Circulation Research, 118(1), 157–172. 26837746 10.1161/CIRCRESAHA.115.305364 PMC4742348

[eph70345-bib-0018] Hyngstrom, A. S. , Murphy, S. A. , Nguyen, J. , Schmit, B. D. , Negro, F. , Gutterman, D. D. , & Durand, M. J. (2018). Ischemic conditioning increases strength and volitional activation of paretic muscle in chronic stroke: A pilot study. Journal of Applied Physiology, 124(5), 1140–1147. 29420152 10.1152/japplphysiol.01072.2017 PMC6050199

[eph70345-bib-0019] Hyngstrom, A. S. , Nguyen, J. N. , Wright, M. T. , Tarima, S. S. , Schmit, B. D. , Gutterman, D. D. , & Durand, M. J. (2020). Two weeks of remote ischemic conditioning improves brachial artery flow mediated dilation in chronic stroke survivors. Journal of Applied Physiology, 129(6), 1348–1354. 33090908 10.1152/japplphysiol.00398.2020 PMC7792845

[eph70345-bib-0020] Ivey, F. M. , Ryan, A. S. , Hafer‐Macko, C. E. , & Macko, R. F. (2011). Improved cerebral vasomotor reactivity after exercise training in hemiparetic stroke survivors. Stroke; A Journal of Cerebral Circulation, 42(7), 1994–2000. 10.1161/STROKEAHA.110.607879 21636819

[eph70345-bib-0021] Jaramillo‐Torres, M. J. , Limpert, R. H. , Butak, W. J. , Cohen, K. E. , Whitaker‐Hilbig, A. A. , Durand, M. J. , Freed, J. K. , & SenthilKumar, G. (2025). Promoting resiliency to stress in the vascular endothelium. Basic & Clinical Pharmacology & Toxicology, 136(3), e70001. 39936288 10.1111/bcpt.70001 PMC12822311

[eph70345-bib-0022] Kempf, K. S. , Whitaker, A. A. , Lui, Y. , Witte, E. , Perdomo, S. J. , Ward, J. L. , Eickmeyer, S. , Ledbetter, L. , Abraham, M. , & Billinger, S. A. (2019). The effect of stroke on middle cerebral artery blood flow velocity dynamics during exercise. Journal of Neurologic Physical Therapy, 43(4), 212–219. 31449179 10.1097/NPT.0000000000000289 PMC6744289

[eph70345-bib-0023] Kirkman, D. L. , Stock, J. M. , Shenouda, N. , Bohmke, N. J. , Kim, Y. , Kidd, J. , Townsend, R. R. , & Edwards, D. G. (2023). Effects of a mitochondrial‐targeted ubiquinol on vascular function and exercise capacity in chronic kidney disease: A randomized controlled pilot study. American Journal of Physiology‐Renal Physiology, 325(4), F448–F456. 37560769 10.1152/ajprenal.00067.2023

[eph70345-bib-0024] Linder, B. A. , Stute, N. L. , Hutchison, Z. J. , Barnett, A. M. , Tharpe, M. A. , Kavazis, A. N. , Kirkman, D. L. , Gutierrez, O. M. , & Robinson, A. T. (2024). Acute high‐dose MitoQ does not increase urinary kidney injury markers in healthy adults: A randomized crossover trial. American Journal of Physiology‐Renal Physiology, 326(1), F135–F142. 37942539 10.1152/ajprenal.00186.2023 PMC11198989

[eph70345-bib-0025] Liu, Y. , Bubolz, A. H. , Mendoza, S. , Zhang, D. X. , & Gutterman, D. D. (2011). H_2_O_2_ is the transferrable factor mediating flow‐induced dilation in human coronary arterioles. Circulation Research, 108(5), 566–573. 21233456 10.1161/CIRCRESAHA.110.237636 PMC3108183

[eph70345-bib-0026] Maeda, H. , Matsumoto, M. , Handa, N. , Hougaku, H. , Ogawa, S. , Itoh, T. , Tsukamoto, Y. , & Kamada, T. (1993). Reactivity of cerebral blood flow to carbon dioxide in various types of ischemic cerebrovascular disease: Evaluation by the transcranial Doppler method. Stroke; A Journal of Cerebral Circulation, 24(5), 670–675. 10.1161/01.str.24.5.670 8488521

[eph70345-bib-0027] McNamara, K. F. , Merkler, A. E. , Freeman, J. V. , Krumholz, H. M. , Ahmad, T. , & Sharma, R. (2024). Ischemic stroke and reduced left ventricular ejection fraction: A multidisciplinary approach to optimize brain and cardiac health. Stroke; A Journal of Cerebral Circulation, 55(6), 1720–1727. 10.1161/STROKEAHA.123.045623 38660813

[eph70345-bib-0028] Miller, K. B. , Howery, A. J. , Rivera‐Rivera, L. A. , Johnson, S. C. , Rowley, H. A. , Wieben, O. , & Barnes, J. N. (2019). Age‐related reductions in cerebrovascular reactivity using 4D flow MRI. Frontiers in Aging Neuroscience, 11, 281. 31680935 10.3389/fnagi.2019.00281 PMC6811507

[eph70345-bib-0029] Moncion, K. , Rodrigues, L. , De Las Heras, B. , Noguchi, K. S. , Wiley, E. , Eng, J. J. , MacKay‐Lyons, M. , Sweet, S. N. , Thiel, A. , Fung, J. , Stratford, P. , Richardson, J. A. , MacDonald, M. J. , Roig, M. , & Tang, A. (2024). Cardiorespiratory fitness benefits of high‐intensity interval training after stroke: A randomized controlled trial. Stroke; A Journal of Cerebral Circulation, 55(9), 2202–2211. 10.1161/STROKEAHA.124.046564 39113181

[eph70345-bib-0030] Murphy, M. P. , & Smith, R. A. (2007). Targeting antioxidants to mitochondria by conjugation to lipophilic cations. Annual Review of Pharmacology and Toxicology, 47, 629–656. 10.1146/annurev.pharmtox.47.120505.105110 17014364

[eph70345-bib-0031] Murphy, S. , Durand, M. , Negro, F. , Farina, D. , Hunter, S. , Schmit, B. , Gutterman, D. , & Hyngstrom, A. (2019). The Relationship between blood flow and motor unit firing rates in response to fatiguing exercise post‐stroke. Frontiers in Physiology, 10, 545. 31133877 10.3389/fphys.2019.00545 PMC6524339

[eph70345-bib-0032] Murphy, S. A. , Negro, F. , Farina, D. , Onushko, T. , Durand, M. , Hunter, S. K. , Schmit, B. D. , & Hyngstrom, A. (2018). Stroke increases ischemia‐related decreases in motor unit discharge rates. Journal of Neurophysiology, 120(6), 3246–3256. 30379629 10.1152/jn.00923.2017 PMC6337044

[eph70345-bib-0033] Park, S. Y. , Pekas, E. J. , Headid, R. J. , 3rd, Son, W. M. , Wooden, T. K. , Song, J. , Layec, G. , Yadav, S. K. , Mishra, P. K. , & Pipinos, I. I. (2020). Acute mitochondrial antioxidant intake improves endothelial function, antioxidant enzyme activity, and exercise tolerance in patients with peripheral artery disease. American Journal of Physiology. Heart and Circulatory Physiology, 319(2), H456–H467. 32706261 10.1152/ajpheart.00235.2020

[eph70345-bib-0034] Qin, L. , Tong, F. , Li, S. , & Ren, C. (2024). Beyond pharmacology: The biological mechanisms of remote ischemic conditioning in cerebrovascular disease. Biomolecules, 14(11), 1408. 39595584 10.3390/biom14111408 PMC11592304

[eph70345-bib-0035] Rossman, M. J. , Santos‐Parker, J. R. , Steward, C. A. C. , Bispham, N. Z. , Cuevas, L. M. , Rosenberg, H. L. , Woodward, K. A. , Chonchol, M. , Gioscia‐Ryan, R. A. , Murphy, M. P. , & Seals, D. R. (2018). Chronic supplementation with a mitochondrial antioxidant (MitoQ) improves vascular function in healthy older adults. Hypertension, 71(6), 1056–1063. 29661838 10.1161/HYPERTENSIONAHA.117.10787 PMC5945293

[eph70345-bib-0036] Weston, M. E. , Barker, A. R. , Tomlinson, O. W. , Coombes, J. S. , Bailey, T. G. , & Bond, B. (2022). The effect of exercise intensity and cardiorespiratory fitness on the kinetic response of middle cerebral artery blood velocity during exercise in healthy adults. Journal of Applied Physiology, 133(1), 214–222. 35708705 10.1152/japplphysiol.00862.2021 PMC9291408

[eph70345-bib-0037] Whitaker, A. A. , Aaron, S. E. , Chertoff, M. , Brassard, P. , Buchanan, J. , Nguyen, K. , Vidoni, E. D. , Waghmare, S. , Eickmeyer, S. M. , Montgomery, R. N. , & Billinger, S. A. (2024). Lower dynamic cerebral autoregulation following acute bout of low‐volume high‐intensity interval exercise in chronic stroke compared to healthy adults. Journal of Applied Physiology, 136(4), 707–720. 38357728 10.1152/japplphysiol.00635.2023 PMC11286270

[eph70345-bib-0038] Whitaker, A. A. , Aaron, S. E. , Kaufman, C. S. , Kurtz, B. K. , Bai, S. X. , Vidoni, E. D. , Montgomery, R. N. , & Billinger, S. A. (2022). Cerebrovascular response to an acute bout of low‐volume high‐intensity interval exercise and recovery in young healthy adults. Journal of Applied Physiology, 132(1), 236–246. 34882027 10.1152/japplphysiol.00484.2021 PMC8759972

[eph70345-bib-0039] Whitaker, A. A. , Waghmare, S. , Montgomery, R. N. , Aaron, S. E. , Eickmeyer, S. M. , Vidoni, E. D. , & Billinger, S. A. (2023). Lower middle cerebral artery blood velocity during low‐volume high‐intensity interval exercise in chronic stroke. Journal of Cerebral Blood Flow and Metabolism, 44(5), 627–640. 37708242 10.1177/0271678X231201472 PMC11197145

[eph70345-bib-0040] Whitaker‐Hilbig, A. A. , Nguyen, J. N. , Wietrzny, A. , Merkow, G. , Tarima, S. , Klevenow, E. , Nelson, L. , Hyngstrom, A. S. , & Durand, M. J. (2025). Effects of ischemic conditioning on microvascular reactivity to single passive limb movement in young adults: A pilot study. European Journal of Applied Physiology, 125(6), 1653–1663. 39984737 10.1007/s00421-025-05717-1 PMC12302722

[eph70345-bib-0043] Whitaker‐Hilbig, A. , Nguyen, J. , Wietrzny, A. , Wietrzny, S. , Desai, A. , Hyngstrom, A. , & Durand, M. (2026). Ischemic conditioning improves microvascular reactivity to single passive limb movement in the paretic leg of chronic stroke survivors. Physiology, 41(S1), 2300236.

[eph70345-bib-0041] Zhao, W. , Hausenloy, D. J. , Hess, D. C. , Yellon, D. M. , & Ji, X. (2023). Remote ischemic conditioning: Challenges and opportunities. Stroke; A Journal of Cerebral Circulation, 54(8), 2204–2207. 10.1161/STROKEAHA.123.043279 37417240

